# Microstructure and Mechanical Properties of Butt Joints between Stainless Steel SUS304L and Aluminum Alloy A6061-T6 by TIG Welding

**DOI:** 10.3390/ma11071136

**Published:** 2018-07-04

**Authors:** Van Nhat Nguyen, Quoc Manh Nguyen, Shyh-Chour Huang

**Affiliations:** 1 Department of Mechanical Engineering, National Kaohsiung University of Science and Technology, 415, Chien-Kung Road, Sanmin District, Kaohsiung 80778, Taiwan; duynhat240685@gmail.com; 2 Department of Mechanical Engineering, Hung Yen University of Technology and Education (UTEHY), Khoai Chau 39000, Hung Yen, Vietnam; manhrobocon@gmail.com

**Keywords:** TIG welding, A6061 alloy, SUS304L steel, ER4047 filler metal, IMC, butt joints

## Abstract

The tungsten inert gas (TIG) welding method most commonly used to weld ferrous metals, nonferrous metals, and other metals since it is simple, easily implemented, and achieves consistent high-quality welds. In this study, butt joints produced between aluminum alloy A6061-T6 and stainless steel SUS304L have been achieved by using TIG welding with ER4047 filler metal. The macrostructure and microstructure of the resulting specimens were analyzed by means of an optical microscope (OM), a scanning electron microscope (SEM), and an energy dispersive X-ray spectrometer (EDS). A uniform intermetallic layer was found at the interface between the stainless steel and the weld seam having a thickness of 2 µm, and the intermetallic compound (IMC) includes Fe_4_Al_13_, Fe_2_Al_5_, and FeAl_3_ phases. The micro-hardness and tensile strength of the weld joints were also investigated. Due to Si content in the compensating metal, there was a prevention of iron diffusion into the aluminum, thus hindering the development of the IMC layer and reducing its thickness in such a way that the weld joint strength increases. The analyzed results show that the average micro-hardness of the stainless steel, weld seam, aluminum alloys, and IMC layer were 218 HV, 88.3 HV, 63.3 HV, and 411 HV, respectively. The fracture occurred at the brazed interface, and the ultimate tensile strength value reached 225 MPa.

## 1. Introduction

In order to improve diverse product attributes such as product quality, weight reduction, cost reduction and savings, and decreased environmental pollution during the industrial welding applications, a hybrid structure of dissimilar metals including Al/Mg, Al/Cu, Al/Ti, Al/Al, Ti alloy/Fe alloy, Mg/Fe [1,2,3,4,5,6,7,8] has been adopted for industrial usage. For example, joints between aluminum alloy and stainless steel are commonly used because of their acceptable strength, low density, and exceptional corrosion resistance. However, it should be noted that the obtainment of good quality welding joints between aluminum and steel represents a considerable challenge because of their significant relative differences in their mechanical properties, physical properties, and melting temperatures. Specifically, iron–aluminum intermetallic compound (IMC) layers easily form hardened and brittle interface surface areas between the weld and the stainless steel side, which greatly reduces the strength of welded joint [9,10,11]. Therefore, in order to ameliorate this circumstance, many different welding methods have been utilized for the welding of aluminum alloys and stainless steel, including friction stir welding [4,12], metal inert gas welding [13,14,15,16,17], laser welding [5,18,19,20,21], resistance sport welding [22], and ultrasonic welding [23,24]. Although, these methods are not without their limitations, namely, the cost of specialized welding equipment is exorbitant, the limited shape structure of the welding components, and the exclusive weld locations due to space restrictions.

By comparison, the tungsten inert gas (TIG) welding method is simple, easy to accomplish, the price of welding equipment is acceptable, welds are seldom restricted due to space limitations, and the weld temperature is controllable. Therefore, the TIG welding method is the most commonly form of welding for different material applications. For example, Song et al. [25] have analyzed the effects of Si on the IMC layer during dissimilar butt TIG of AISI 321 stainless steel and aluminum alloy 5A06 with an application of three kinds of filler metals. They pointed out that the content of Si that was added prevented the Fe diffusion into the liquid, somewhat reducing the growth of IMC layer. The mechanical properties of the interlayer are optimized with a Si content of 5%. Similarly, Lin et al. [26] investigated the microstructure and mechanical properties of butt joining-brazing between 5A06 aluminum alloy and SUS 321 stainless steel by using TIG welding with Al-Cu6 filler metal non-corrosive flux. These researchers found that the IMC layer appeared at the interface between the weld seam and the steel side, with a thickness of 3–5 µm. In another work, Lin et al. [27] also found two intermetallic phases which formed at the interface between the weld seam and the steel, with the weld side having a phase of τ_5_–Al_7_Fe_2_Si, and the stainless steel side having a phase of θ-FeAl_3_. When these researchers studied the metallurgical and mechanical properties of dissimilar tungsten inert gas-welded butt joints between aluminum alloy 5A06 to stainless steel SUS 321 utilizing BJ380A filler wire and modified non-corrosive flux. Research revealed that the mechanical properties of the butt joints obtained in this procedure, such as an incidence of tensile strength measured at 125 MPa, while the highest hardness value of 950 HV was attained. Ye et al. [28] compared the microstructural and mechanical properties which occurred during the butt joint welding of AA 5052 aluminum alloy and Q235 low-carbon steel produced by Metal Inert Gas-Tungsten Inert Gas (MIG-TIG) double-sided arc welding-brazing and the weld obtainable through a MIG welding tradition process. Their results proved that the double-sided welding-brazing method produced a good welding appearance at relatively lower welding temperatures. The intermetallic layer obtained in such an operation is thinner on average, while the average tensile strength is 2.5 times greater when compared with traditional MIG welding. Besides, Shao et al. [29] chose to examine the microstructure of joints for aluminum and steel under the influence of pulsed double-electrode gas metal arc (Pulsed DE-GMA) welding-brazing parameters. They reported that a reduction in the bypass current leads to a decrease in the intermetallic layer thickness. In addition, they pointed out that there are two phases, namely Fe_2_Al_5_ and FeAl_3_, to be found in the IMC layer of the weld. It should be noted that there have been no published studies to date on the possibility of welding aluminum alloy with stainless steel SUS304L. In this study, dissimilar A6061-T6 alloy and SUS304L steel butt joints were successfully produced by means of a pulse tungsten inert gas (TIG) welding-brazing process, and the microstructural and mechanical properties of the weld joint were discussed.

## 2. Experimental Details

**Figure 1:**
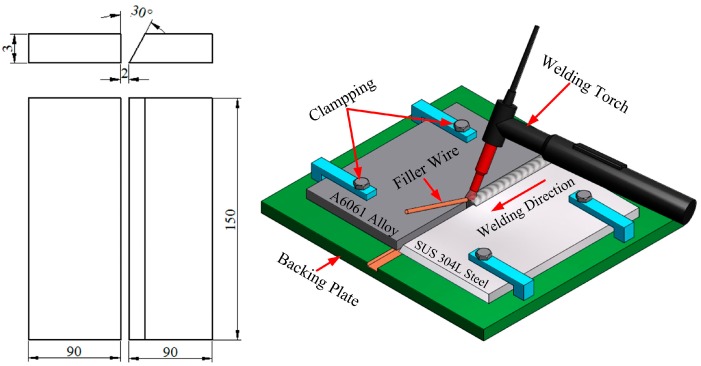
Schematic illustrations of Al/steel tungsten inert gas (TIG) joint.

Joining between dissimilar materials has been used extensively in a number of industrial applications such as in the automotive industry, aerospace industry, and shipbuilding industry [30,31,32,33]. Base metals including aluminum alloy A6061-T6 sheet and stainless steel SUS304L plate were used for purposes of this study, with both dimensions of 150 mm × 90 mm × 3 mm, along with a single bevel-joint preparation maintaining a bevel angle of 30° on the steel side. The process of the beveling of stainless steel sheets was carried out by means of an X6332B milling machine. The entire process of preparation for the welding joint was carried out in accordance with American Welding Society standard (AWS D1.6/D1.6M) [34]. The filler metal was ER 4047 (Al-12%Si) with the diameter of 1.6 mm. Argon shield gas with a 99.99% purity was chosen to protect the weld and to avoid oxidation. The nominal compositions and mechanical properties of the parent materials and filler wire are shown in Table 1, Table 2 and Table 3 [35,36,37]. In order to reduce any possible distortions generated after welding, the base metal plates were fixed onto the exposed surface of the sub-plate. Before welding, any grease or oil residue, along with the surface oxide film of the materials, was removed by manually scrubbing the materials with a steel brush. The Syncrowave 250DX welding machine (Miller Electric Manufacturing Co., Spencer Street Appleton, WI, USA) used in this welding procedure successfully performed a sample weld, with the optimal welding parameters being set as a welding current of 60 A, a welding voltage of 21 V, a welding speed of 6 mm/s, and a gas flow rate of 14 L/min. The butt-joint configuration and diagram of the introduced welding process are shown in Figure 1.

**Figure 2:**
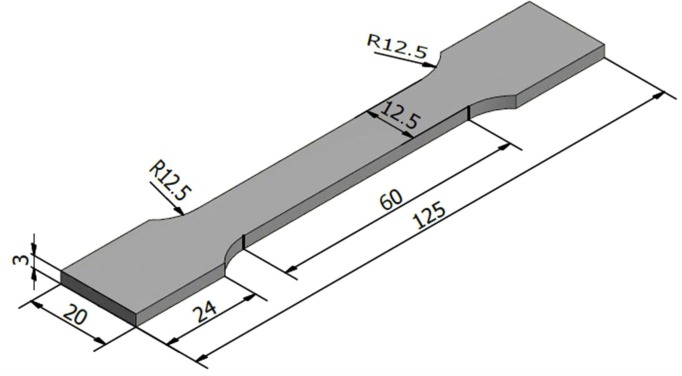
The geometry and dimensions of the tensile-test sample (mm).

After welding was completed, a typical cross section of the welds was cut perpendicular to the welded line by means of a wire cutter A3017. Once obtained, the cross-section of the welds was fixed in plastic with a casting time of 30 min, a casting temperature of 1200 °C, and a press of 786 kPa. Next, the welding samples were ground and polished by means of a Mopao 1000 automatic grinder/polisher machine, with metallographic sandpaper grits of 450, 600, 800, 1200, 1500, 2000 SiC grades, and buffed using Al_2_O_3_ particles up to 0.05 µm. Then, the surface of the sample was cleaned with 5 mL of a HNO_3_ + 100 mL C_2_H_5_OH solution at room temperature for 4 s. An optical microscope (*Nikon Eclipse ME600*, Nikon, Tokyo, Japan) was used to investigate the macro-structure of the interface formed between the weld seam and the steel. The micro-structure and composition of the IMC were observed by means of a scanning electron microscope (SEM) and an energy dispersive JSM-7000F X-ray spectroscope (EDS), and an X-ray was made to determine phase formation in both the IMC and weld zones. Finally, the mechanical properties of the welded joint have been characterized by their micro-hardness and tensile strength, and the tensile-test specimen was pointed out in Figure 2.

## 3. Results and Discussion

### 3.1. Macrostructure and Weld Appearance

**Figure 3:**
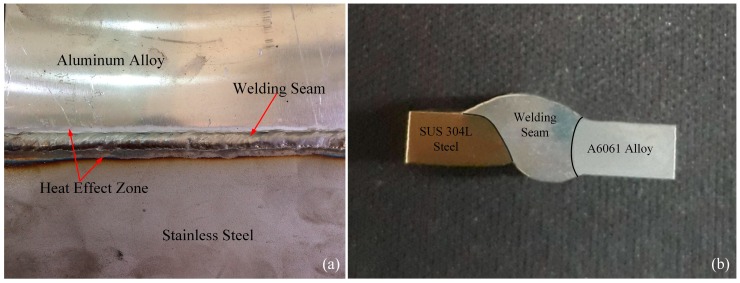
Appearance of the weld joint (**a**) and cross-section (**b**) of the specimen.

The surface and cross-section of the TIG weld is displayed in Figure 3. With optimum welding parameters selected, the surface appearance of the welded joint is good, smooth, sans metal splashing, of uniform weld width, and without apparent defects such as cracking, undercutting, and porosity. Figure 3a reveals that the heat-affected zone appeared in both the aluminum and steel sides, and the width of the heat-affected zone has increased towards the end of the welding seam. The heat-affected area of the steel side is larger than that aluminum side because during welding, the temperature of the arc is directly angled towards the steel side. While, further observation of the weld specimen’s cross-section in the Figure 3b indicates that the fusion zone between the stainless steel and aluminum alloy sides has been distinguished by black lines. In the welding process, the arc’s elevated temperature melts the filler wire and the aluminum alloy to form the welding fusion. With the SUS304L steel side, the brazing interface layer was formed owing to the reaction of the stainless steel surface and the molten filler metal.

### 3.2. Microstructure

**Figure 4:**
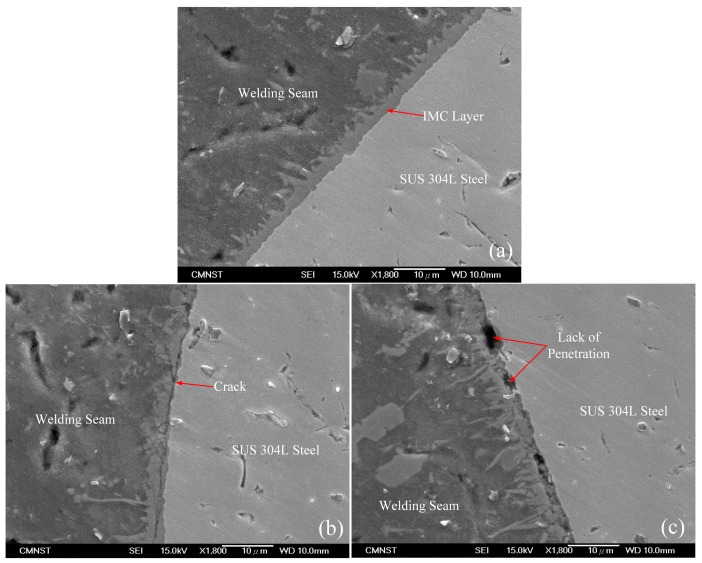
Scanning electron microscope (SEM) image of Al-steel joint; (**a**) intermetallic compound (IMC) layer zone; (**b**) Crack defect; (**c**) Lack of penetration defect.

A scanning electron microscope process was carried out in order to investigate the characteristics of the braze interface, with the analyzed results are shown in Figure 4. Figure 4a reveals that a thin uniform IMC layer has formed along the weld joint, with the ICM layer thickness being 2 µm; it is smaller than the limited value of 10 µm. This result is due in large part to the presence of the Si element in the filler metal, which prevents the diffusion of Fe atoms into the molten aluminum, and reduces the overall development of the IMC layer entirely. Figure 4a indicates that according to the direction to the weld-seam side, the IMC layer has a lath-shape; and, according to the direction to the stainless steel, the IMC layer has more of a whisker shape. Therefore, the interface of the IMC layer is unsmooth, portraying a wave-shaped orientation toward the welded seam, while it is extremely smooth along the stainless-steel side. The inter-layer sample obtained in this study is thinner than other studies. Such as, Song et al. [25] examined the micro-structural and mechanical properties of the solder joints between 5A06 aluminum alloy and AISI 321 stainless steel by TIG welding with different filler metals. They demonstrated that the IMC layer thickness increases 6–8 µm when the weld was made by means of ER4047 filler wire. Zhang et al. [17] had researched MIG lap-joining of aluminum alloy 2B50 and stainless steel 1Cr18Ni9T with ER4043 filler rod. They illustrated that the IMC layer thickness changed 5–15 µm. Further observations exhibited the existence of cracks and a lack of penetration defects in the brazed interface, as shown in Figure 4b,c, respectively. The occurrence of cracks is due to the differential cooling rate in the aluminum and steel. These defects will have an adverse effect on the mechanical properties of the weld if they were to be deemed acceptable.

**Figure 5:**
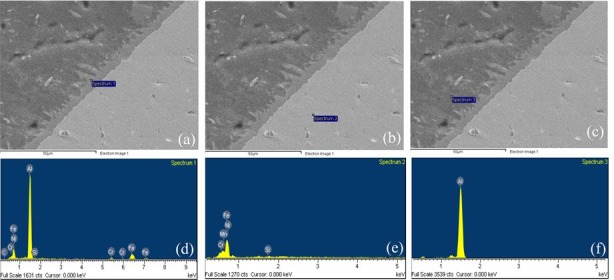
The energy dispersive X-ray spectrometer (EDS) images of TIG weld. (**a**) SEM image of spectrum1; (**b**) SEM image of spectrum2; (**c**) SEM image of spectrum3; (**d**) the distribution of alloying elements at spectrum1; (**e**) the distribution of alloying elements at spectrum2; (**f**) the distribution of alloying elements at spectrum3.

**Figure 6:**
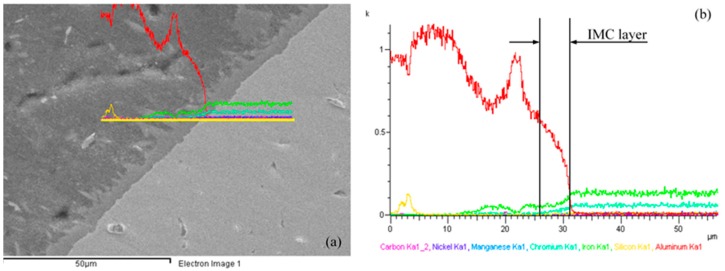
(**a**) Position of linear scanning; (**b**) the corresponding line scan result.

**Figure 7:**
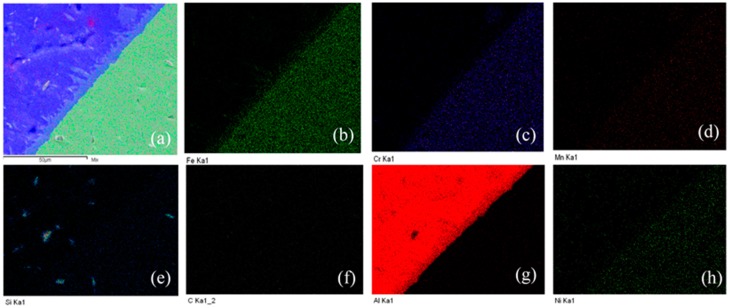
Mapping image and distribution of alloying elements for the A6061 and SUS304L joint. (**a**) SEM mapping image; (**b**) the distribution of Fe element; (**c**) the distribution of Cr element; (**d**) the distribution of Mn element; (**e**) the distribution of Si element; (**f**) the distribution of C element; (**g**) the distribution of Al element; (**h**) the distribution of Ni element.

In order to find the composition of the elements and their distribution in a welded joint produced between aluminum and steel, EDS elemental analysis and linear scanning were applied, as displayed in Figure 5 and Figure 6. Figure 5a–c points out the locations of the EDS elemental test and, Figure 5d–f shows the distribution of major alloying elements in the weld. Results are revealed in Table 4. The composition found at the IMC layer (Spectrum 1) was 1.86 at. % C; 62.62 at. % Al; 0.35 at. % Si; 1.32 at. % Ni; 3.86 at. % Cr; and, 29.98 at. % Fe. The composition found at the stainless steel side (Spectrum 2) consists of 0.57 at. % Si; 8.69 at. % Ni; 19.13 at. % Cr; 70.94 at. % Fe; 0.67 at. % Mn; 99.70 at. % Al; and, 0.30 at. % Fe in the weld seam (Spectrum 3). The EDS analysis results also denote that the concentration of aluminum alloy at Spectrum 1 is the highest, followed by the content of the Fe element, with the content of Si being the lowest. With a very low concentration, the Si element cannot participate in the formation of a ternary compound, but it is still relatively easy to make solid solutions in the IMC layer. This proves that the Si element contained in the filler metal prevents the diffusion of Fe into the weld, which suppressed the development of the IMC layer and improves the tensile strength of the welding joints. The similar results have been shown in other studies [25,38]. Figure 6 shows that the EDS linear scanning analysis results of the butt joint formed between A6061-T6/SUS304L using the pulse TIG welding process utilizing ER4047 filler wire. The analysis results pointed out that the content of Al decreased via the direction from the welding seam to the steel side, while the Si and Fe elements gradually increased, and Al was combined with Fe to form IMC layers AlxFey. Figure 7 shows the results of the EDX elemental mapping of the elements (Al, Si, Fe, Mn, Cr) on the cross section of the welded sample.

**Figure 8:**
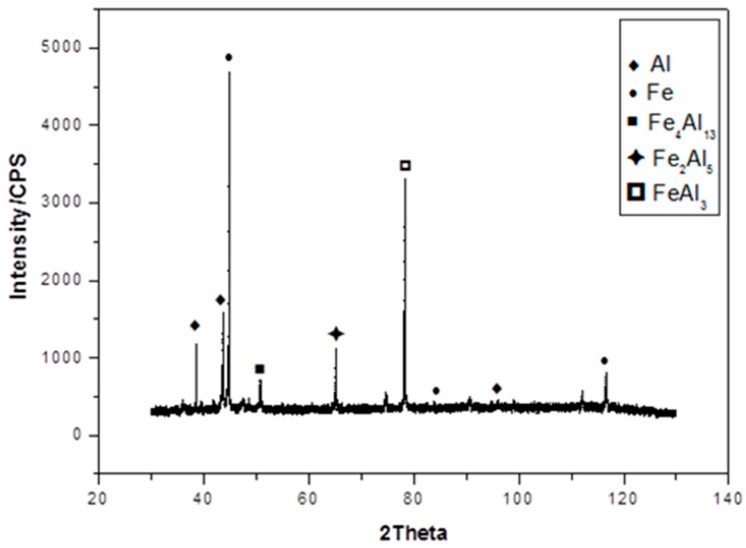
XRD analysis results of Al/Steel specimen.

The X-ray diffraction test method was applied to identify the phase composition of the IMC layer, as shown in Figure 8. The new phases formed in the IMC layer included Fe_4_Al_13_, Fe_2_Al_5_, and FeAl_3_ phase. FeAl_3_ is an equivalent phase with the α(Al) phase, and can usually be represented with Fe_4_Al_13_ and Fe_2_Al_7_. The IMC’s layer nearest the steel side is pointed out to be FeAl_2_ and Fe_2_Al_5_, and the IMC’s layer nearest the weld seam is indicated to be Fe_4_Al_13_. The formation of these phases can be explained as follows. During the arc welding process, the temperature of the arc column is directed toward the stainless steel plate, which immediately matches the temperature of the arc heating the Fe atoms, and gives the Fe atoms large amounts of energy to diffuse into a short, rather confined distance. Further, the temperature of the welding arc continues to melt the filler wire and aluminum alloy basic to form a liquid metal pool, then, this liquid spreads onto the surface of stainless steel sheet. At this time, the Fe atoms in the solid phase combine with the Al atoms in the liquid phase to produce phase Fe_2_Al_5_. However, due to the relatively quick cooling rate of the molten pool and overall welding speed, it makes the Fe atoms in the Fe_2_Al_5_ phase partially crystallize to form the FeAl phase. Finally, the FeAl_3_ phase continues to combine with aluminum atoms in order to form the Fe_4_Al_13_ phase.

**Figure 9:**
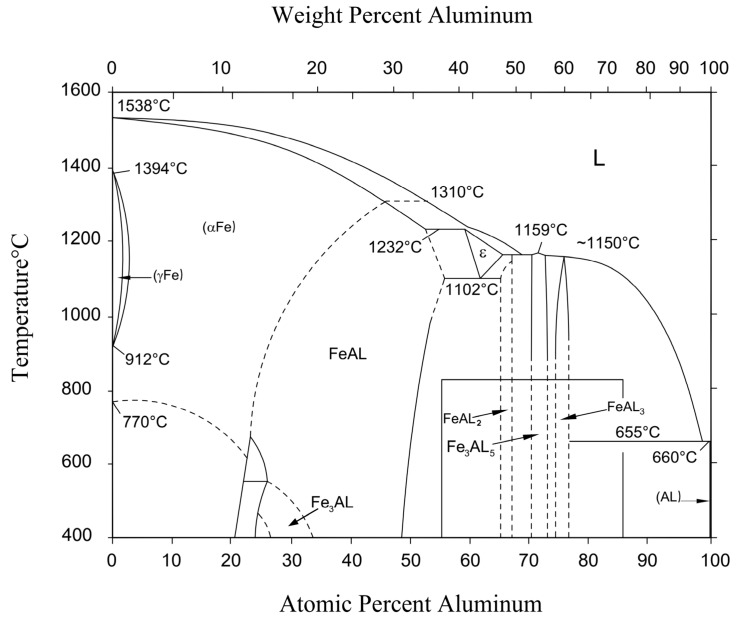
The Fe-Al binary phase diagram.

In addition, the formation of new phases in the IMC layer may also be determined in the binary phase diagram of the Fe-Al [39], as denoted in Figure 9. There are five new phases that can be formed in the IMC layer, including the Fe_3_Al, FeAl, FeAl_2_, Fe_2_Al_5_, and FeAl_3_ phases.

### 3.3. Mechanical Properties

**Figure 10:**
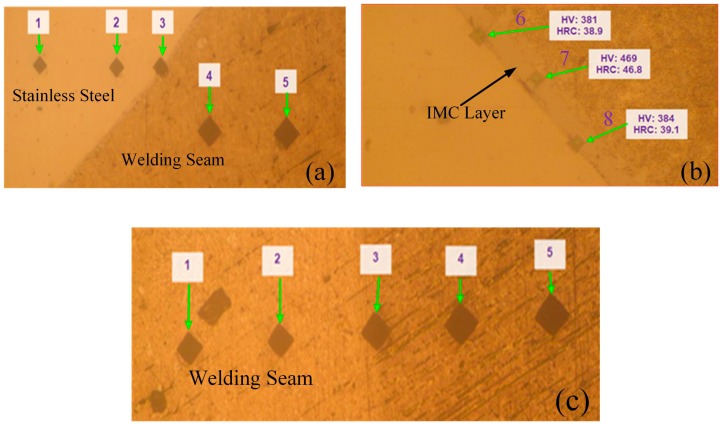
The micro-hardness tester position; (**a**) from stainless steel to welding seam; (**b**) intermetallic compound; (**c**) from welding seam to aluminum alloys.

The mechanical properties of the welded joints in this study are characterized by micro-hardness and tensile strength. Vickers hardness testing was performed on the specimen’s cross-section with a 10 N loading force, and held for 10 s to measure the micro-hardness from the SUS304L steel side to the welding seam, and from the welding seam to the aluminum alloys side, especially at the IMC layer, as shown in Figure 10. The measurement results revealed that the hardness values at the locations are non-uniform. The average hardness values of the stainless steel zone, weld metal zone, aluminum alloys zone, and intermetallic compound layer were 218, 88.3, 63.3 and 411 HV, respectively. The maximum hardness value measured at the 7 position was 469 HV, because in the IMC layer contained the hard Fe_4_Al_13_ phase. This result has indicated that the average hardness value at the welding seam is greater than the mean hardness value measured on the aluminum alloys side and lower on the stainless steel side. The hardness value measured at the welding area is higher than that of the aluminum alloys due to the diffusion of the alloying elements from the filler wire into the weld pool.

After welding, the weld joints are always stronger than the base metals in both tensile strength and surface shape of weld joints, they are important parameters that can be used to evaluate the quality of solder joints. When the weld joints were putting into use in different environments, the temperature (the temperature conditions can be increase or decrease) will effects and causing difficulties to components weld joints. Therefore, a tensile strength testing of weld joints will give the information and the characteristics of the joints after welding. The specimens were tested at room temperature conditions. Five samples selected from 9 welding samples for the tensile strength test were welded under the same conditions. The results of the tensile tests of the five welded specimens are presented in Table 5. As the results in Table 5 show, the maximum strength of the welds was achieved at 225 MPa, and the average was 208.4 MPa. The average value of the specimens was higher than the tensile strength of the welding rod ER4047 at 60%, and this average value was approximately 73% of the tensile strength of the A6061-T6 alloys. The results exhibited that the largest tensile strength of the test specimens is also higher than the tensile strength of the welding rod ER4047, and approximately 79% of the tensile strength of the A6061-T6 alloy, as Liu, H et al. [35]. The results indicated that good quality joints were obtained.

**Figure 11:**
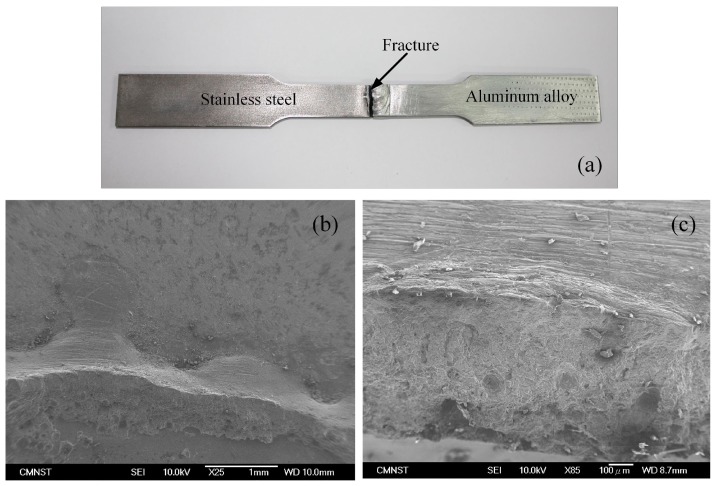
The result of the tensile test process; (**a**) Fracture position; (**b**) Fracture surface at stainless steel side; (**c**) Fracture surface at aluminum side.

The fracture surface of the weld specimen was illustrated in Figure 11 is perpendicular to the direction of load action, and it occurs at the welding brazing surface. Fracture characteristics is a combination of brittle fracture and cleavage fracture. The location where the fault occurs is the weakest position in the weld joint, because this position was contained an IMC layer and micro-cracks. During the tensile test, initial cracks appeared at the root of the weld, and then propagated along the interface between the welding seam and the stainless steel to form the fault line. The fracture surface has been shown very clearly in Figure 11b,c. Figure 11b shows the fault surface on the steel side, and Figure 11c exhibits to the fault surface at the aluminum side. The SEM morphology of the fractured surface is not smooth; it consists of many dimples. Especially, there is the occurrence of a splitting phenomenon in this area.

## 4. Conclusions

Based on the analysis results of the microstructure and mechanical properties of the butt joint produced between A6061-T6 alloy and SUS304L steel using the tungsten inert gas welding method with ER4047 filler metal, the main summary is indicated as follows.

- Dissimilar metals weld between an A6061-T6 aluminum alloy and SUS304L stainless steel were done by tungsten inert gas welding–brazing with welding rod ER4047. The welding joint had a good formation with no defects such as cracks, porosity, and undercut, which appeared on the weld surface. In addition, the size of the heat-affected zone was very small.
- The Si element prevented the diffusion of Fe into the weld pool, and limited the formation and growth of the interlayer. As a result, a thin intermetallic compound layer was formed along the interface between the welded joint and the stainless steel with a thickness of 2 µm, and it was smaller than the thickness limit. Phase compositions found in the interlayer contained Fe_4_Al_13_, Fe_2_Al_5_, and FeAl_3_ phases.
- The mechanical properties of the welded joint depend on the thickness of the brittle intermetallic compound layer and the formation of microscopic cracks inside the welding seam. The quality of the weld joints was improved when the thickness of the intermetallic compound layer was minimum.
- The micro-hardness value gradually decreased from the stainless steel to the welding seam, and the average hardness value of the SUS304L steel was found to be 218 HV, the welding seam was 88.3 HV, the aluminum alloys side was 63.3 HV, and the intermetallic compound layer was 411 HV.
- The ultimate tensile strength of the test specimens obtained was higher than the tensile strength of the filler metal ER 4047, and it was approximately 79% compared tensile strength of the A6061-T6 aluminum alloy. The fault locations occurred at the interface between the welding seam and the steel. The fracture process of the tensile specimen started from the micro-cracks located on the Fe_2_Al_5_ brittle phase. Here, the material’s cleavage occurred and created a fracture. The fracture model of welding joint was a combination of the brittle fracture and the cleavage fracture.

## Figures and Tables

**Table 1:** Nominal compositions and mechanical properties of A6061-T6 aluminum alloy.

Nominal Compositions (wt %)									Mechanical Properties		
Al	Fe	Si	Cr	Mg	Ti	Cu	Mn	Zn	Ultimate tensile strength (MPa)	Yield strength (MPa)	Elongation (%)
Bal.	<0.7	0.4–0.8	0.04–0.35	0.8–1.2	<0.15	0.15–0.4	<0.15	<0.25	284	240	14

**Table 2:** Nominal compositions and mechanical properties of SUS304L.

Nominal Compositions (wt %)								Mechanical Properties		
C	Mn	Si	P	S	Cr	Ni	N	Ultimate Tensile Strength (MPa)	Yield Strength (MPa)	Elongation (%)
0.03	2.0	0.75	0.045	0.03	19.5	12.0	0.10	485	170	40

**Table 3:** Nominal compositions and mechanical properties of filler wire.

Nominal Compositions (wt %)									Mechanical Properties		
Al	Si	Fe	Cu	Mn	Mg	Zn	Ti	Be	Ultimate Tensile Strength (N/mm^2^)	Yield Strength (N/mm^2^)	Elongation (%)
Bal.	11.0–13.0	<0.60	<0.30	<0.15	<0.1	<0.20	<0.15	0.0003	130	60	5

**Table 4:** Chemical Compositions of different points at the cross-section of the Al/Steel weld joint.

Locations	Elements wt %						
	C	Al	Si	Ni	Cr	Fe	Mn
Spectrum 1	1.86	62.88	-	1.33	3.87	30.06	-
Spectrum 2	-	-	0.57	8.69	19.13	70.94	0.67
Spectrum 3	-	99.70	-	-	-	0.30	-

**Table 5:** Results of tensile test of five specimens’ welded A6061-T6 alloys to SUS304L stainless steel.

Samples	T-1	T-2	T-3	T-4	T-5	Average	ER4047	A6061-T6
Values (MPa)	218	192.5	208.5	225	196.2	208.4	130	284
